# Rapid and specific biotin labelling of the erythrocyte surface antigens of both cultured and *ex-vivo Plasmodium *parasites

**DOI:** 10.1186/1475-2875-6-66

**Published:** 2007-05-22

**Authors:** Lisa Sharling, Kordai MP Sowa, Joanne Thompson, Helen M Kyriacou, David E Arnot

**Affiliations:** 1 Institute of Immunology and Infection Research, School of Biological Sciences, University of Edinburgh, King's Buildings, Edinburgh, EH9 3JT, Scotland, UK; 2 Centre for Tropical and Emerging Global Diseases, University of Georgia, 729 Biological Sciences Building, Athens, GA 30602, USA; 3 Centre for Medical Parasitology, University of Copenhagen, CSS-Øster Farimagsgade 5, Building 23, PO Box 2099,1014 Copenhagen, Denmark

## Abstract

**Background:**

Sensitive detection of parasite surface antigens expressed on erythrocyte membranes is necessary to further analyse the molecular pathology of malaria. This study describes a modified biotin labelling/osmotic lysis method which rapidly produces membrane extracts enriched for labelled surface antigens and also improves the efficiency of antigen recovery compared with traditional detergent extraction and surface radio-iodination. The method can also be used with *ex-vivo *parasites.

**Methods:**

After surface labelling with biotin in the presence of the inhibitor furosemide, detergent extraction and osmotic lysis methods of enriching for the membrane fractions were compared to determine the efficiency of purification and recovery. Biotin-labelled proteins were identified on silver-stained SDS-polyacrylamide gels.

**Results:**

Detergent extraction and osmotic lysis were compared for their capacity to purify biotin-labelled *Plasmodium falciparum *and *Plasmodium chabaudi *erythrocyte surface antigens. The pellet fraction formed after osmotic lysis of *P. falciparum-infected *erythrocytes is notably enriched in suface antigens, including PfEMP1, when compared to detergent extraction. There is also reduced co-extraction of host proteins such as spectrin and Band 3.

**Conclusion:**

Biotinylation and osmotic lysis provides an improved method to label and purify parasitised erythrocyte surface antigen extracts from both *in vitro *and *ex vivo Plasmodium *parasite preparations.

## Background

After erythrocyte invasion, *Plasmodium *merozoites differentiate and modify the host cell plasma membrane to increase nutrient uptake [1-5] and also, at least in *Plasmodium falciparum*, to modify its adhesion properties [6-8]. Parasite proteins on the infected erythrocyte (IE) surface, often referred to as parasitized erythrocyte surface antigens (PESAs) include the *P. falciparum *erythrocyte membrane protein-1 (PfEMP1) family, the rifin protein family [9,10] and other less well-characterized antigens, such as the recently identified proteins; parasite-IE surface proteins 1 and 2 (PIESP 1 and 2) [11]. PfEMP1s and rifins are encoded by multi-gene families, the *var *and *rif *families, respectively. It has been proposed that certain clinical malarial syndromes are caused by cytoadhesion phenotypes mediated by the expression of particular subsets of the *var *multi-gene family. However demonstrating that expression of a specific *var *gene/PfEMP1 protein causes a particular adhesion phenotype remains experimentally challenging.

Identification and purification of PESAs is difficult, due to the low abundance of these antigens and the lack of specific reagents to identify particular antigen variants [12,13]. Using high-energy radioisotopes for surface labelling has become more difficult in many Western research facilities and is beyond the capacity of most field-based clinical laboratories with access to fresh, patient-derived material for pathological analyses. Therefore the studies were carried out using a biotin labelling/osmotic lysis method that can rapidly produce membrane extracts enriched for labelled surface antigens. The N-hydroxysuccinimide (NHS) ester vitamin biotin (sulpho-NHS-LC-biotin) reacts efficiently with primary amino groups and primarily labels lysine residues and the N-termini of proteins. Rifin and PfEMP1 proteins typically contain around 10% lysine residues (averaged from current sequence data) making these proteins good targets for labelling. Although sulpho-NHS-LC-biotin is internalized by IE through the parasite's novel permeation pathway (NPP), Baumeister et al. [14] have shown that NPP inhibitors such as furosemide [4,15] prevent the uptake of sulpho-NHS-LC-biotin and subsequently the biotinylation of internal proteins. However in this study it was not clear whether sulpho-NHS-LC-biotin can efficiently label PESAs.

This study demonstrates that the exposure of intact IEs to sulpho-NHS-LC-biotin in the presence of furosemide and the extraction of labelled proteins by osmotic lysis provides an alternative to traditional radioisotope labelling and detergent-based protein extraction methods. This method was also used to specifically label erythrocyte surface antigens from *ex vivo *samples of the rodent malaria parasite *P. chabaudi *and thus can be applied to the labelling of *ex vivo P. falciparum *clinical isolates in a field laboratory setting to aid in understanding the relationship between clinical malaria syndromes and parasitized erythrocyte surface antigen expression.

## Methods

### Biotin surface labelling of *Plasmodium *infected erythrocytes

*P. falciparum *clone R29, previously selected to display the rosetting phenotype was cultured using standard conditions and synchronised by sorbitol treatment. *P. chabaudi *(clone AS) infected erythrocytes were obtained from infected CBA mice at peak asexual parasitaemia. Mice were bled, serum was removed and the infected erythrocytes placed into short term *in vitro *culture for six hours to allow late stage parasites to mature and express erythrocyte surface antigens. Material was then processed as described below.

3 × 10^6 ^erythrocytes from *P. falciparum in vitro *cultures (8–10% parasitaemia) or *ex vivo P. chabaudi *short term cultures (40–50% parasitaemia) and uninfected controls were washed twice with phosphate buffered saline (PBS, pH 7.2). Trypsin treated cells were incubated with 1.0 mg/ml trypsin-TPCK (Worthington Biochemicals) in a final volume of 1.0 ml in PBS, for 10 minutes at 37°C. 'Mock' treated (non-protease treated) cells were incubated with PBS alone. The reaction was terminated by adding soybean trypsin inhibitor (Worthington Biochemicals) to a final concentration of 1 mg/ml and washed twice in PBS before further use.

Cell pellets were re-suspended in 10 volumes of in PBS/200 μM furosemide/0.6 mM CaCl_2_/1 mM MgCl_2 _for 1 minute prior to the addition of sulpho-NHS-LC-biotin (Pierce) to a final concentration of 0.5 mg/ml. To prevent hydrolysis of sulpho-NHS-LC-biotin prior to use sulpho-NHS-LC-biotin was stored in sealed aliquots to minimise exposure moisture. Surface biotinylation was then carried out for 30 minutes at room temperature, with gentle agitation. The biotinylation reaction was stopped by pelleting the cells and resuspending the pellet in PBS/200 μM furosemide/0.6 mM CaCl_2_/1 mM MgCl_2_/100 mM glycine. Cells were washed again with PBS/200 μM furosemide/0.6 mM CaCl_2_/1 mM MgCl_2_. The integrity of infected erythrocytes after surface biotinylation was confirmed by microscopy.

### Detergent extraction of biotin labelled proteins

Surface biotinylated erythrocytes were lysed on ice with five volumes of a solution containing 150 mM NaCl, 5 mM EDTA, 50 mM Tris pH8.0, 1.0% Triton-X100 (w/v) and protease inhibitor cocktail (Roche). Cell lysates were centrifuged at 13,000 rpm at 4°C for 10 minutes, the Triton-soluble fraction (Ts) was removed and the Triton-insoluble pellet (Ti) re-suspended in a solution of 2% (w/v) SDS, 20 mM Tris-HCl pH 8.0, 150 mM HCl. High molecular weight parasite DNA in the SDS- solubilized Ti fraction was sheared by repeated pipetting and the SDS-soluble supernatant (SDS-Ti) was removed after the samples were again centrifuged at 13,000 rpm for 10 minutes at 4°C.

### Protein extraction by osmotic lysis

Osmotic extracts were prepared after surface biotinylation by lysing erythrocytes on ice with five volumes of ice-cold 5 mM Na_2_HPO_2 _(pH 8.0) containing a protease inhibitor cocktail (Roche). Cell lysates were then centrifuged at 13,000 rpm for 10 minutes at 4°C. The supernatant (Os) and erythrocyte membrane layer (Om) were removed to fresh tubes, and the membrane layer was washed twice with ice-cold 5 mM Na_2_HPO_2 _(pH 8.0) containing protease inhibitors. The remaining pellet (Op) was treated with the endonuclease benzonase (Novagen) by resuspending the pellet in 60 μl of Bugbuster™ protein extraction reagent (Novagen) containing 30 units of the endonuclease. Non-infected erythrocytes were given identical treatment as the infected erythrocytes. No Op was recovered after osmotic lysis of non-infected erythrocytes.

### Western blotting and silver staining

SDS-PAGE and Western blotting were performed using the NuPAGE pre-cast gel system (Invitrogen) using 3–8% Tris-acetate gels following the manufacturer's instructions. Electrophoretic transfer of proteins to polyvinylidene difluoride membrane (Schleicher & Schuell BioScience) was carried out using a transfer buffer containing 1.25 mM bicine, 1.25 mM bis-tris, 0.05 mM EDTA (pH 7.2) and 10% methanol. A commercially available non-biological blocking reagent (QIAGEN) was used prior to chemiluminescent detection of biotinylated proteins with 0.02 μg/ml horseradish peroxidase-conjugated streptavidin (Pierce Biotechnology) and antibody incubations. The PfEMP1-ATS peptide antibody [16] was detected using horseradish peroxidase-conjugated protein A (Pierce Biotechnology) diluted to a final concentration of 2.5 μg/ml.

Silver staining was performed by sequential incubation in the following solutions: (1) 40% ethanol/10% acetic acid for 30 minutes; (2) 30% ethanol/160 mM sodium thiosulphate/490 mM sodium acetate for 30 minutes; (3) distilled water for 3, 5 minute washes; (4) 15 mM silver nitrate for 20 minutes; (5) distilled water for 2, 1 minute washes; (6) 236 mM sodium carbonate (Sigma)/0.75% (w/v) formaldehyde for 2–5 minutes; (7) 40 mM EDTA for 10 minutes.

## Results

### The fate of biotin labelled *P. falciparum *erythrocyte surface antigens following either detergent extraction or extraction by osmotic lysis

**Figure 1:**
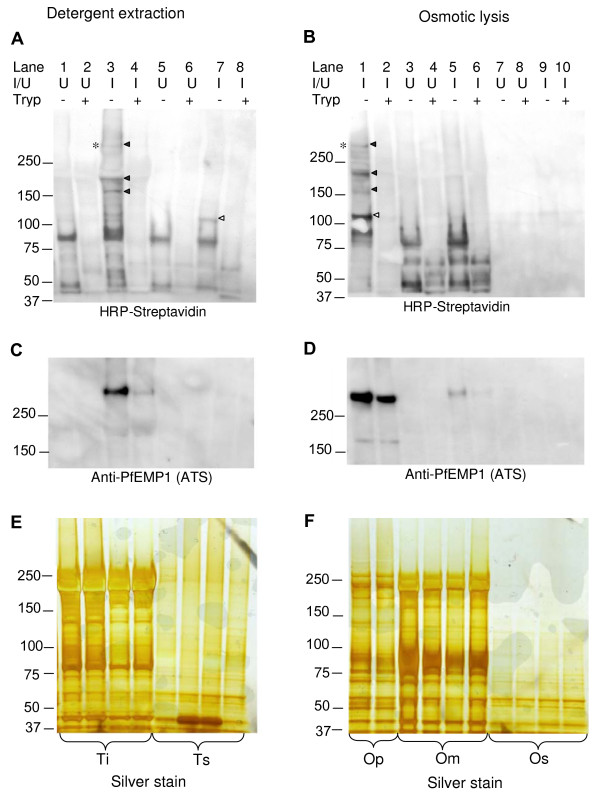
**Detergent and osmotic lysis extracts of biotinylated *P. falciparum *PESAs**. **Panel A**. Western blotting of detergent extracts from surface biotinylated *P. falciparum *infected erythrocytes. Biotinylated proteins were detected by horseradish peroxidase-linked streptavidin. **Panel B**. Western blotting of surface biotinylated *P. falciparum *infected erythrocyte extracts prepared by osmotic lysis with biotinylated proteins detected by horseradish peroxidase-linked streptavidin. **I/U **indicates extracts from *P. falciparum *infected and uninfected erythrocytes. The first four lanes in panels A, C and E contain Triton X-100 insoluble material (**Ti**) whereas the succeeding four lanes contain Triton X-100 soluble material (**Ts**). The first two lanes in panels B, D and F contain the insoluble fractions which can be pelleted by centrifugation after osmotic lysis (**Op**), the next four lanes contain the post-osmotic lysis membranous fraction (**Om**) and the final two lanes contain the osmotic lysis supernatant fraction (**Os**). The solid arrowheads highlight Triton-insoluble, infected erythrocyte-specific, trypsin-sensitive biotinylated proteins. The empty arrowhead highlights a ~110 kDa Triton-soluble, infected erythrocyte-specific, trypsin-sensitive biotinylated protein which also partitions into the osmotic lysis pellet.**Panel C **and **D**. Blots A and B respectively, were re-probed with an anti-PfEMP1 antiserum to show co-localization with a biotin-labelled, infected erythrocyte-specific, trypsin-sensitive protein expressed by the R29 parasite clone. **Panel E **and **F**. Silver stained duplicate gels of the gels used to prepare the Western blots shown in Panel A and B respectively to confirm the integrity of the protein extracts.

A number of infected erythrocyte specific proteins were biotin labelled (Figure 1A &1B) and enriched in cellular fractions prepared by either detergent extraction (Figure 1A, C &1E) or osmotic lysis (Figure 1B, D &1F). *P. falciparum *IE-specific biotin-labelled proteins were sensitive to surface trypsin treatment (Figure 1A, lanes 4 and 8; Figure 1B, lane 2), indicative of exposure on the IE surface. Several high molecular weight *P. falciparum *IE-specific biotin-labelled proteins exhibited detergent solubility characteristics of PfEMP-1, i.e. they were insoluble in 1% Triton-X-100, but soluble in 2% SDS (Figure 1A, Lane 3). To determine if any of the IE-specific biotinylated proteins co-localised with the PfEMP1 expressed by R29 parasites the blots were stripped of HRP-streptavidin and re-incubated with affinity purified rabbit antiserum [16] raised against a relatively well conserved peptide sequence (DITSSESEYEELDINDIC) in the acidic terminal sequences of all PfEMP1 proteins currently in Genbank. One trypsin-sensitive, biotin-labelled protein of >250 kDa co-localised with a band that reacted with the anti-PfEMP-1 antiserum (Figure 1C, lane 3 and Figure 1D, lane 1) and consistent with the previous finding that the R29 *P. falciparum *clone when selected for a rosetting phenotype expresses the R29varl *var *gene (Accession No. CAA73831) which encodes a 308 kDa PfEMP1 [17]. The proteins reacting with the known anti-PfEMP-l antiserum were present in very low abundance in the trypsinized samples (Figure 1C, lane 4) indicating that the detergent is, as expected, extracting surface exposed PfEMP-1.

By contrast, the majority of PfEMP1 extracted by osmotic lysis into the insoluble pellet (Op) fraction was trypsin insensitive (Figure 1D, lane 2) indicating that this extraction method yields an insoluble precipitate containing relatively large amounts of PfEMP1 that is not surface exposed and thus insensitive to proteases. This trypsin-resistant fraction of PfEMP1 presumably derives from the intracellular pool in transit to the cell surface. Importantly, although osmotic lysis yields intracellular PfEMP1, a surface exposed population of PfEMP1 is also present in this fraction (Fig 1B, lanes 1 & 2). In addition the trypsin-sensitive, 110 kDa protein extracted by Triton-X100 was also present in the osmotic lysis pellet (Op) (Figure 1A, lane 7; Figure 1B, lane 1). The osmotic lysis pellet fraction thus contains relatively larger amounts of PfEMP1 than are present in detergent extracts, some additional biotin-labelled parasite-specific high molecular weight PESAs and a 110 kDa Triton-soluble protein. The osmotic lysis pellet contains primarily parasite nuclei and insoluble material. No pellet fraction was recovered after osmotic lysis of non-infected erythrocytes.

Duplicate gels of those shown in Figures 1A and 1B were silver-stained to confirm that trypsin-sensitivity was a specific feature of surface exposed antigens (Figures 1E and 1F). The silver staining also reveals that a significant portion of host-cell derived proteins, such as spectrin and Band 3, remain in the osmotic lysis erythrocyte membrane fraction (Figure 1F, lanes 2 and 3) and that the osmotic lysis insoluble pellet fraction consequently contains much less 'contaminating' spectrin and Band 3 proteins when compared to detergent extracts (Figure 1E).

### Biotinylation of *P. chabaudi *erythrocyte surface antigens and extraction by osmotic lysis

**Figure 2:**
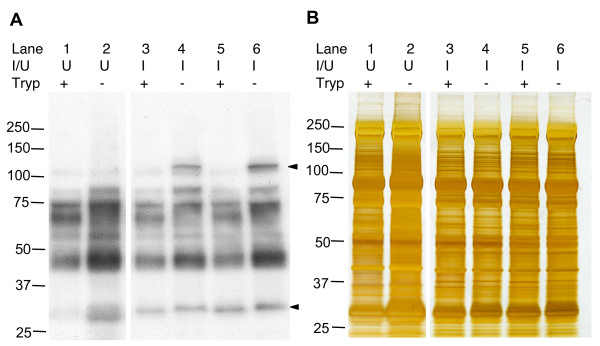
**Western blot analysis of biotinylated *P. chabaudi *PESAs**. *P. chabaudi *infected erythrocytes were obtained from two infected CBA mice by heart puncture and cultured for 6 hours to allow ring stage parasites to mature. **Panel A**. Western blotting of *P. chabaudi *infected erythrocyte membrane extracts prepared by osmotic lysis. Biotinylated proteins were detected using horseradish peroxidase-conjugated streptavidin. Solid arrowheads highlight infected erythrocyte-specific biotinylated proteins. **Panel B**. Silver stained duplicate gel of that shown in Panel A to confirm the cell surface specificity of trypsinization. **U**: uninfected erythrocyte, **I**: infected erythrocyte.

Osmotic lysis extraction of surface biotinylated *P. chabaudi *infected erythrocytes enriched for a parasite-specific biotinylated trypsin-sensitive protein of ~110 kDa in the membrane fraction (Figure 2A). A protein with the same characteristics was detected in three further AS-derived *P. chabaudi *clones. An additional trypsin-sensitive protein of ~30 kDa, the approximate size of the previously described cir proteins [18], was biotin-labelled in the *P. chabaudi *AS strain (Figure 2A). Extracts from uninfected erythrocytes contained a biotinylated protein that ran at a slightly lower molecular of 30 kDa. The biotinylated portions of this protein however were completely removed by trypsin-treatment as it was absent in extracts from trypsin treated uninfected erythrocytes. The ~30 kDa protein in uninfected extracts also ran as a diffuse band as opposed to the ~30 kDa biotinylated protein in IE extracts which repeatedly ran as a compact band and although a small mobility shift was apparent following exposure to trypsin, its intensity did not change. As is the case for all the parasite-specific biotinylated proteins, without immunoprecipitation it is not possible to discern whether they are parasite derived or IE-surface associated host serum factors.

## Discussion

Two methods, detergent extraction and osmotic lysis, were compared for their capacity to extract biotin-labelled *P. falciparum *PESAs. Pellet fractions formed after osmotic lysis were significantly enriched in PESAs, including PfEMP1, when compared to the detergent extraction procedure, thus providing an easily produced enriched extract for further analyses. The relative enrichment of PESAs by the osmotic lysis method compared to detergent extraction was the result of a reduction in the amount of co-extracted host proteins, such as spectrin and Band 3, and the precipitation of both intracellular and surface exposed pools of PfEMP1. During mass spectrometric analyses of PESA-containing cell fractions, abundant spectrin-derived proteolytic-fragments are readily detected thus reducing the relative abundance of PESA proteolytic-fragments. Osmotic lysis provides an advantageous method to increase the signal-to-noise ratio for subsequent mass spectrometric analysis of biotin-labelled PESA extracts. Capturing the entire cellular pool of PfEMP1 would facilitate subsequent proteomic analysis, as PfEMP1 is not highly expressed and only a fraction of the cellular pool ultimately appears on the erythrocyte surface [19]. Recent proteomic studies using high-throughput multi-dimensional protein identification technology (MuDPIT) found inconsistent developmental expression patterns for PfEMP1 and did not obtain good sequence coverage for this protein [11]. The low abundance of PfEMP1 contributes to the difficulties of carrying out reproducible proteomic studies with this important antigen and therefore a reproducible extraction method that maximizes the PfEMP1 yield is clearly desirable.

In this study 3–4 biotin-labelled surface exposed proteins were found to be specific to *P. falciparum *infected erythrocytes, of which an ~110 kDa protein has not been described in previous studies using radio-iodination [20,21]. This could be explained by sulpho-NHS-LC-biotin's predominant reaction with lysine (rather abundant in PfEMP1) as opposed to the lacto-peroxidase catalysed radio-iodination of tyrosine (rather uncommon in PfEMP1). It is possible that the 110 kDa protein is related to the PIESP1 protein, a PESA recently reported by Florens *et al*. [11]. Their MuDPIT analysis of extracts enriched for surface biotinylated proteins identified two novel *P. falciparum *PESAs, PIESP 1 and 2, but did not detect PfEMP1 or rifins. However secreted proteins (Exp-1 and 2) and rhoptry proteins (RAP 1 and 2, and RhopH 2 and 3) were detected in their analysis. This suggests that the cell surface biotinylation conditions used, which lacked a permeation pathway inhibitor such as furosemide, may not have been surface specific. Smaller proteins with characteristics of the rifin family were not detected by surface-biotinylation in this study. The cause of the lack of rifin labelling is unclear, as this protein family contains a relatively high proportion of lysine residues [22] and thus was anticipated to label well with sulpho-NHS-LC-biotin. Low rifin protein expression levels due to the absence of *in vivo-type *selection pressure during long-term *in vitro *culture and possible masking by co-migrating host erythrocyte surface proteins may contribute to limiting our ability to detect these proteins.

Applying the surface biotinylation-osmotic lysis methodology to the rodent malaria parasite *P. chabaudi *permitted detection of parasite-infected erythrocyte proteins with molecular weights of ~110 kDa and ~30 kDa. The 110 kDa protein was also detected in extracts from two genetically distinct *P. chabaudi *clones. It is larger than the predicted molecular weights of any of the members of the predicted *P. chabaudi *erythrocyte surface antigen multi-gene families described by Fischer *et al*. [18]. However the smaller 30 kDa antigen detected has the characteristics of a cir protein. *Cir *genes encode 30–40 kDa proteins which belong to the ubiquitous *Plasmodium *interspersed repeat (*pir*) super-family, members of which have been found in several rodent, human (the *rif *genes) and primate malarias [23]. Biotinylation and osmotic lysis appear to give good PESA-labelling results following short-term culture of *P. chabaudi *infected erythrocytes obtained *ex vivo *from mouse infections. This technique may thus be useful for direct identification of the adhesion phenotypes of circulating *P. falciparum *parasites from patients suffering from defined severe malaria syndromes, without the loss of specific variant antigen expression that occurs in long-term culture.

## Conclusion

Biotinylation in the presence of permeation pathway inhibitors, followed by osmotic lysis is a rapid and relatively simple surface specific labeling procedure for *Plasmodium *erythrocyte surface antigens. It provides an estimate of the molecular weight of enriched biotin-labeled proteins, considerably refines the starting protein mixture for proteomic analyses and potentially could be used to analyze the surface protein expression on *ex vivo *clinically derived parasites.

## Authors' contributions

LS conceived of the study, maintained *P. falciparum *culture, and performed the experiments with the assistance of HMK. KMPS and JT participated in the design of the study. DA helped fund the study and write the manuscript. All authors read and approved the final manuscript.
